# Transcriptome analysis of human cancer reveals a functional role of Heme Oxygenase-1 in tumor cell adhesion

**DOI:** 10.1186/1476-4598-9-200

**Published:** 2010-07-28

**Authors:** Stefanie Tauber, Alexander Jais, Markus Jeitler, Sandra Haider, Julia Husa, Josefine Lindroos, Martin Knöfler, Matthias Mayerhofer, Hubert Pehamberger, Oswald Wagner, Martin Bilban

**Affiliations:** 1 Department of Laboratory Medicine, Medical University of Vienna, Vienna, Austria; 2 Department of Clinical Pharmacology, Medical University of Vienna, Vienna, Austria; 3 Department of Obstetrics and Fetal-Maternal Health, Reproductive Biology Unit, Medical University of Vienna, Vienna, Austria; 4 Department of Dermatology, Medical University of Vienna, Vienna, Austria

## Abstract

**Background:**

Heme Oxygenase-1 (HO-1) is expressed in many cancers and promotes growth and survival of neoplastic cells. Recently, HO-1 has been implicated in tumor cell invasion and metastasis. However, the molecular mechanisms underlying these biologic effects of HO-1 remain largely unknown. To identify a common mechanism of action of HO-1 in cancer, we determined the global effect of HO-1 on the transcriptome of multiple tumor entities and identified a universal HO-1-associated gene expression signature.

**Results:**

Genome-wide expression profiling of Heme Oxygenase-1 expressing versus HO-1 silenced BeWo choriocarcinoma cells as well as a comparative meta-profiling of the preexisting expression database of 190 human tumors of 14 independent cancer types led to the identification of 14 genes, the expression of which correlated strongly and universally with that of HO-1 (P = 0.00002). These genes included regulators of cell plasticity and extracellular matrix (ECM) remodeling (MMP2, ADAM8, TGFB1, BGN, COL21A1, PXDN), signaling (CRIP2, MICB), amino acid transport and glycosylation (SLC7A1 and ST3GAL2), estrogen and phospholipid biosynthesis (AGPAT2 and HSD17B1), protein stabilization (IFI30), and phosphorylation (ALPPL2). We selected PXDN, an adhesion molecule involved in ECM formation, for further analysis and functional characterization. Immunofluorescence and Western blotting confirmed the positive correlation of expression of PXDN and HO-1 in BeWo cancer cells as well as co-localization of these two proteins in invasive extravillous trophoblast cells. Modulation of HO-1 expression in both loss-of and gain-of function cell models (BeWo and 607B melanoma cells, respectively) demonstrated a direct relationship of HO-1 expression with cell adhesion to Fibronectin and Laminin coated wells. The adhesion-promoting effects of HO-1 were dependent on PXDN expression, as loss of PXDN in HO-1 expressing BeWo and 607B cells led to reduced cell attachment to Laminin and Fibronectin coated wells.

**Conclusions:**

Collectively, our results show that HO-1 expression determines a distinct 'molecular signature' in cancer cells, which is enriched in genes associated with tumorigenesis. The protein network downstream of HO-1 modulates adhesion, signaling, transport, and other critical cellular functions of neoplastic cells and thus promotes tumor cell growth and dissemination.

## Background

Heme oxygenases are the rate-limiting enzymes in heme degradation that catalyze the conversion of heme into carbon monoxide, iron, and biliverdin. Heme oxygenase 1 (HO-1) has (cyto)protective properties and antiinflammatory, antiapoptotic, and antiproliferative capacities of HO-1 have been described in several cell types [1,2]. Under normal physiologic conditions HO-1 expression is low but can be upregulated in response to a wide range of stimuli and activated signaling molecules, including the HO-1 substrate heme, reactive oxygen species (ROS), nitric oxide species, prostaglandins, cytokines, growth factors such as insulin, and lipopolysaccharide [2]. Since heat shock (and other cellular stressors) lead to upregulation of HO-1, this molecule has also been termed heat-shock protein 32 (Hsp32).

A relation between malignant behavior and alterations in expression of HO-1 may exist. Elevated HO-1 has been detected in several cancer cell lines [3-6] and tumors (including lymphosarcoma, adenocarcinoma, hepatoma, glioblastoma, melanoma, prostate cancers, Kaposi sarcoma, squamous carcinoma, pancreatic cancer, brain tumors and myeloid leukemias; reviewed in [7]), thereby affecting tumor cell apotosis, proliferation, invasion and metastasis [7]. Furthermore, HO-1 gene polymorphisms have been associated with increased cancer susceptibility [8,9].

Cell adhesion is an important determinant of organised growth and the maintenance of architectural integrity. Changes in cell-cell and cell-extracellular matrix (ECM) adhesion accompany the transition from benign tumours to invasive, malignant cancers and the subsequent metastatic dissemination of tumour cells [6,10,11]. Specifically, alterations in ECM remodeling have been shown to affect adhesion properties of neoplastic cells. Although several studies have linked expression of HO-1 with various stages of tumor progression [12-15], the molecular mechanisms underlying HO-1-mediated changes in adhesion of neoplastic cells remain elusive.

We used gene expression profiling as a global assay to identify a common gene set directly linked to HO-1 in 14 cancer types. One of the genes that emerged was PXDN, the human homologue of the *Drosophila *gene peroxidasin. PXDN is a cell surface peroxidase associated with the extracellular matrix [12] and was found to play a key role in HO-1-dependent cell adhesion of neoplastic cells in our investigations. Our results reflect, for the first time, that HO-1 mediates genome-wide effects on transcriptional regulation of genes potentially involved in tumorigenesis. Moreover, our findings provide insights into the mechanisms underlying HO-1-dependent tumor invasion and support the notion that HO-1 represents a molecular target in cancer.

## Materials and metods

### Construction of transgenic cell lines

**Figure 1:**
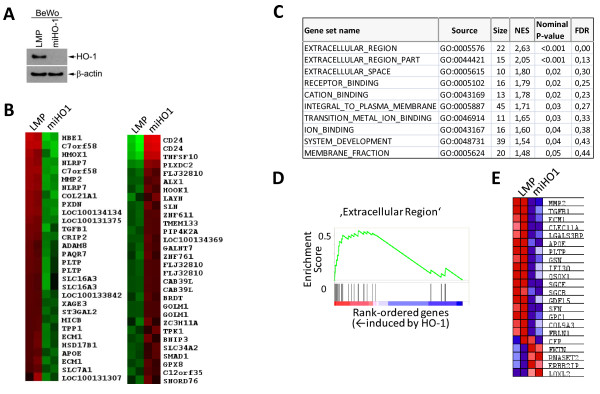
**The HO-1 gene signature in BeWo cancer cells**. (A) Western blot analysis of ctrl (LMP) or HO-1 shRNAmir-transduced BeWo cells (miHO-1) demonstrates efficient HO-1 knock-down. β-actin indicates equal loading. A representative example is shown. (B) A heat map of top 30 genes that discriminate HO-1 expressing (LMP) from HO-1 knockdown (miHO-1) cells as judged by LIMMA analysis (p < 0.05). Downregulated genes are shown in green and upregulated genes in red. Note that some genes are represented by multiple probe sets on the GeneChip. (C) Pathways enriched in HO-1 expressing BeWo cells. The number of genes contained within one pathway/gene set is given by 'Size'. Normalized enrichment score ('NES') is calculated by the GSEA software. Statistical significance is indicated by the nominal P-value ('NOMp-val') and the error is controlled by the false discover rate ('FDR q-val'). (D) Distribution of 20 pathway molecules for the top-ranking gene set amid the total ranked list of all transcripts analyzed by GSEA. Genes were rank ordered based on differential expression between LMP and miHO1 BeWo cells. (E) Corresponding heatmap showing relative expression of the 'Extracellular Region' pathway gene members in LMP and miHO-1 BeWo cells. Downregulated genes are shown in blue and upregulated genes in red.

Constitutive stable HO-1 knock-down in BeWo choriocarcinoma cells (European Collection of Cell Cultures (Salisbury, UK) was generated by transduction with a microRNA (miRNA) adapted retroviral vector. Briefly, an shRNAmir (microRNA-adapted short hairpin RNA) against human HO-1 in pSM2 vector (oligo ID: V2HS_133107; Open Biosystems, Huntsville, AL, USA) was subcloned into the LMP vector Open Biosystems). Constitutive HO-1 overexpression in 607B melanoma cells [16] (kindly provided by Dr. Volker Wachek, was kindly provided by V. Wacheck; Department of Clinical Pharmacology, Medical University of Vienna, Austria) was generated by transduction with the retroviral vector MSCVpuro (Clontech, Mountain View, CA, USA) containing the human HO-1 cDNA [17]. For production of recombinant retroviruses, HEK293FT cells (Invitrogen, Carlsbad, CA, USA) were co-transfected with a vector containing the viral packaging proteins gag and pol, a vector containing env, and either LMP (ctrl), LMP-miHO1 (LMP containg miRNA against human HO-1), MSCV (ctrl) or MSCV-HO1 (MSCV containing the HO-1 cDNA). Vectors containing gag, env, and pol were kind gifts from Dr. Ewan Rosen (Beth Israel Deaconess Medical Center, Harvard Medical School, Boston , MA, USA). Forty-eight hours after transfection, viral supernatants were collected, BeWo and 607B cells were transduced in the presence of polybrene (8 μg/ml). Stable integrants were selected with puromycin (5 μg/ml). Knock-down or overexpression of HO-1 was verified by Western blotting (Fig 1A and Fig Seven A).

### Transient Transfections

Small interfering (si) RNA targeting human PXDN, negative control siRNA (oligo ID: HSS187890 or cat. no. 12935-200, respectively; Invitrogen), pCDNA 3.1 (Invitrogen), or a plasmid containing the full PXDN cDNA under control of the CMV promoter (clone ID: OCABo5050A058, ImaGenes, Berlin, Germany) )were delivered into BeWo cells by nucleofection (Amaxa, Lonza Bioscinece) according to a previously optimized protocol [18].Briefly, 1 × 10^6 ^BeWo cells were nucleofected with siRNA (100 nmol/L) or 1 μg of control (pcDNA) or pPXDN plasmids following the manufacturers' instructions (solution V, program X-005) (Amaxa Biosystems, Germany). Following transfection, cells were kept in culture for 48-72 hrs, followed by cell adhesion assays.

### Isolation of total RNA and DNA-Microarry expression profiling

BeWo choriocarcinoma cells were purchased from the European Collection of Cell Cultures (ECACC, Salisbury, UK) and were cultured in Ham F12 medium (Gibco Life Technologies, Paisley, UK) supplemented with 5% fetal bovine serum (FBS; Biochrom, AG, Berlin, Germany) and streptomycin/penicillin (Gibco) using standard culture conditions. Total RNA was extracted from subconfluent culture using an RNeasy kit (Qiagen). Total RNA (200 ng) was then used for GeneChip analysis. Preparation of terminal-labeled cDNA, hybridization to genome-wide human Gene Level 1.0 ST GeneChips (Affymetrix, Santa Clara, CA, USA) and scanning of the arrays were carried out according to manufacturer's protocols https://www.affymetrix.com. RMA Signal extraction, normalization and filtering was performed as described (http://www.bioconductor.org/; [19]). A variation filter was applied for selecting informative (i.e., significantly varying) genes. The filtering criteria for the exemplary data sets required an interquantile range > 0.5 and at least one sample with expression intensity > 100. The full gene lists are now available at Gene Expression Omnibus http://www.ncbi.nlm.nih.gov/geo/query/acc.cgi?acc=GSE20404.

### Gene set enrichment analysis (GSEA)

GSEA [20], is a computational method that determines whether a given set of genes (e.g. known pathways, specific areas of the genome or clusters from a cluster analysis) shows statistically significant differences between two phenotypic states (i.e. LMP vs. miHO-1). Briefly, the GSEA calculation involves 3 steps: calculation of an Enrichment Score (ES) followed by estimation of the significance level of ES and adjustment for Multiple Hypothesis Testing. We used a publicly available database of gene sets contained within the Molecular Signature Database (MSigDB; [20]) to test for enrichment upon HO-1 knockdown.

### Statistical Microarray Group Comparisons

To calculate differential gene expression between individual sample groups, we performed a statistical comparison using the LIMMA package as described previously [19]. Briefly, LIMMA estimates the fold change between predefined sample groups by fitting a linear model and using an empirical Bayes method to moderate the standard errors of the estimated log-fold changes for each probe set [21]. A multiple testing correction based on the false discovery rate (FDR) was performed to produce adjusted p-values. All calculations were performed in ''R.''

### Human tumor gene expression databases

Human tumor gene expression data was used from the Global Cancer Map comprising 190 specimens of 14 different tumor types (breast, pancreas, lung, bladder, ovary, melanoma, uterus, renal, prostate, central nervous system, lymphoma, colorectal, mesothelioma, and leukemia)[22]. Gene expression data from the normal tissues were discarded. Only the data related to cancerous tissues were further analyzed. The GeneNeighbors module of the GenePattern software was used to identify genes, the expression of which was closely correlated with that of HO-1 [23]. Heatmap construction: We used the Pearson distance as a measure of similarity in the expression pattern. This algorithm produced a numerical score that represented the calculated Pearson distance for each gene relative to the HO-1 gene. The genes were then ranked so that the low score indicates the close similarity of the expression pattern of the particular gene with that of the HO-1 gene.

### Kolmogorov-Smirnov statistics

To evaluate the significance of the coexpression pattern of genes, we used the Kolmogorov-Smirnov (KS) statistics. For our analysis, we selected the genes that are differentially expressed in LMP vs miHO1 cells with at least a 2-fold difference (i.e. out of 214 differentially expressed genes, 67 genes were coexpressed with HO1, leaving 45 input genes after mapping onto the respective arrays). We discarded the genes with either overly low or overly high expression levels (<50 and >15,000 relative units in more than half of the arrays, respectively). We also did not include genes that had either less than a 2-fold difference or less than a 50 relative unit difference across all tumor tissues. Finally, out of 16063 genes, 7978 remained. We then determined the positional distribution of the 45 genes within the list of 7978 genes ordered by the Pearson distance relative to HO-1 in the 190 tumor tissues and reported the 14 genes (out of the 45 input genes) being closest to HO-1. In other words, we selected the 14 genes displaying the smallest Pearson distance relative to HO-1. These genes are coexpressed with HO-1 in the tumor specimens and also induced by HO-1 in BeWo cells expressing HO-1 endogenously. We next calculated the KS score for these 14 genes using R. The higher the KS score, the more the expression pattern of the particular gene set is analogous across all tumors. We also performed the same KS analysis for 14 randomly selected genes using 100,000 permutations. The frequency of events when the KS score of the randomly chosen gene set was equal to or exceeded that of the target gene set was taken as a P value (P = 0.00002).

### Real-time PCR

Total RNA (1 μg) was reverse transcribed into cDNA by MMLV enzyme (Promega, Mannheim, Germany) with random hexamers (1 μg/μg total RNA). All PCRs were performed using the SYBR Geen kit (BioRad, Hercules, CA, USA). Primers for selected genes were designed using Primer3 software http://frodo.wi.mit.edu/cgi-bin/primer3/primer3_www.cgi with the following sequences,: HO-1 (CAGGATTTGTCAGAGGCCCTGAAGG, fwd; TGTGGTACAGGGAGGCCATCACC, rev) ADAM8 (CCGCTACGTGGAGCTGTATG, fwd; CCAGCATCTGGAACTCTGCAT, rev), COL21A1 (GAACCCTGGCTACCCTGGAC, fwd; GTGTCCCTGCAATTCCCTG, rev), CRIP2 (CGCTGCAGCAAGAAGGTG, fwd; 5' -GCCAATCCTTGCCCAGAG, rev), IFI30 (CCTACGGAAACGCACAGGA, fwd; GAACTCCCACCTGCCACTG , rev), MICB (ACCTCAGGAGGACCCTGACTC , fwd; GGAGGGAATGCAAGCCTC , rev), MMP2 (GACCTTGGGAGAAGGCCAAG , fwd; CCATCGGCGTTCCCATACT , rev), PXDN (GTCGTGGCCCACCTGACTG , fwd; GTGTCGCTGGGAATGCTG , rev), TGFB1 (TGGAGCCTGGACACGCAGTA , fwd; GCCCGGGTTATGCTGGTTG , rev) and ARP (GCCAATAAGGTGCCAGCTGCTG, fwd; TCTTGCCCATCAGCACCACAG, rev). Using the ABI Prism 7700 sequence detection system (PE Applied Biosystems, Warrington, UK), PCR cycling conditions were as follows: initial denaturation at 95°C for 10 min, followed by 40 cycles at 94°C for 30 seconds, 60°C for 15 seconds and 72°C for 30 seconds and a 10 minutes terminal incubation at 72°C. Sequence Detector Software (SDS version 1.6.3, PE Applied Biosystems) was used to extract the PCR data, which were then exported to Excel (Microsoft, Redmond, WA) for further analyses. The RNA-amount of the human Arp gene was used as an internal control. Data were analyzed according to the 2^-ΔΔCT ^method [24].

### Western blot analyses

Western blot analyses were performed using standard protocols as recently done [25]. Equal amounts of protein lysates (35 μg) were separated on 10% SDS/polyacrylamide (PAA) gels and transferred onto Polyvinylidene fluoride (PVDF)-membranes (GE Healthcare, Amersham, Buckinghamshire, UK). After blocking filters were incubated overnight (4°C) with monoclonal mouse antibodies against human HO-1 (clone OSA110; 1:1000; Stressgen, Ann Arbor, MI, USA), PXDN (clone clone A01; 1:1000; Abnova, Taipei City, Taiwan), β-actin (1:5000; Abcam, Cambridge, MA, USA). After 1 h of treatment (room temperature) with secondary antibodies (anti-mouse Ig horseradish peroxidase linked, Amersham; 1:20.000) signals were developed by using ECL Plus Western Blotting Detection System (Amersham Pharmacia Biotech, Piscataway, NJ, USA).

### Immunofluorescene

Cells grown on coverslips were washed, fixed with 4% paraformaldehyde, permeabilized with 0.5% Triton-X 100 and blocked with goat serum. Expression of HO-1 and PXDN were detected with the antibodies described above. Cells were incubated with secondary antibodies conjugated to Alexa594 or Alexa488 (1:500; Molecular Probes, Eugene, OR, USA) and visualized using a Zeiss Axioskop 2 microscope, Zeiss Axiocam and Photoshop. As negative control coverslips were incubated with the respective isotype control IgG and secondary antibodies (data not shown). First trimester placental tissue were dehydrated and embedded in paraffin (Merck) as described elsewhere [26]. Serial sections (2-3 μm) were prepared, deparaffinized and finally heated in a microwave oven (×2 5 min, 850 W). After incubation in blocking solution (NEN, Boston,MA, USA), slides were incubated overnight with primary antibodies, washed 3 times in PBS (each 5 min) and followed by incubation with secondary antibodies conjugated to Alexa594 or Alexa488 (1 hour, Molecular Probes, Eugene, OR). The following primary antibodies/dilutions were utilized: PXDN (Sigma, 1:50) cytokeratin 7 (clone OV-TL, 8,3 μg/ml, DAKO, Glostrup, Denmark), HO-1 (clone OSA-110, 1:1000, Stressgen, Ann Arbor, MI), Ki67 (clone Ki-S5, 10 μg/ml, Chemicon), Kip2/p57 (C-20, rabbit, 2 μg/ml, Santa Cruz Biotechnolgy, Santa Cruz, CA) and Vimentin (clone Vim 3B4, Dako). As a negative control, the primary antibody was replaced by buffer or isotype IgG. Finally, all sections were counterstained with 1 μg/ml DAPI (Roche) and covered with Fluoromount-G (Soutech, Birmingham, AL).

### Cell Adhesion Assay

Adhesion assays were performed as described by [27], with minor modifications. 96 well plates were coated overnight at 4°C with Fibronectin (10 μg/ml; SIGMA), rat tail Collagen , or with Laminin (10 μg/ml; SIGMA) in PBS. Wells were rinsed and blocked for 1 h with 1% BSA in PBS. Logarithmic phase cells were harvested with trypsin and plated at 40 000 cells per well. After 30 min of incubation at 37°C, wells were rinsed to remove non-adherent cells. Adhered cells were fixed in 10% formalin for 5 min and stained with 0.1% crystal violet (in 20% MeOH) for 5 min. Excess dye was washed off with water and absorbance was measured at 595 nm. Bars represent mean absorbance +/- SEM of each condition tested in triplicates. All values have had background substracted that represents cell adhesion to wells blocked with 1% BSA in PBS.

### Cell Invasion assay

The invasion of BeWo cells was measured by using the Transwell chambers (Chemicon, Millipore, CA) according to the manufacturer's protocol. Briefly, the BeWo cells were electroporated with 20 μM of a control siRNA or siRNA targeting human PXDN with the Amaxa method as described elsewhere [18]. 24 hours later, the cells were seeded onto the membrane of the upper chamber of the transwell at a concentration of 2×10^5^/ml in 500 μl of DMEM/F12 medium. The medium in the upper chamber was serum-free. The medium at the lower chamber contained 10% Foetal Calf serum as a source of chemoattractants. Cells that passed through the Matrigel coated membrane were stained with Cell Stain Solution containing crystal violet supplied in the Transwell Invasion assay (Chemicon, Millipore, CA) and photographed after 20 hours of incubation.

### Cell Proliferation Assay

The effect of CO on proliferation of RAECs was determined with a nonradioactive bromodeoxyuridine (BrdU)-based cell-proliferation assay [28](per the manufacturer's guidelines; Roche, Basel, Switzerland). Following electroporation of 1 × 10^6 ^BeWo cells with 20 μM control or PXDN siRNA according to a previously optimized protocol [18], 2500 cells were seeded into 96 well plates and left for 24 hrs to recover. The cells were stimulated to proliferate with 10% FBS and BrdU incorporation was measured at indicated time points.

### Statistical analysis

Student's t test was used for comparison between the groups. P value < 0.05 was considered significant.

## Results

### Gene expression profiling

We used gene expression profiling to determine the genome-wide effect of HO-1 on the transcriptome of BeWo choriocarcinoma cells. BeWo cells were used in these experiments because these cells show relatively high levels of endogenous HO-1 expression. Expression of HO-1 was silenced in BeWo cells by a micro-RNA adapted retroviral vector targeting human HO-1. Western blotting demonstrated an efficient knockdown of HO-1 expression in BeWo cells stably expressing miHO-1 (henceforth referred to as 'miHO-1') as compared to BeWo cells stably expressing the LMP control sequence (referred to as 'LMP') (Fig. 1). RNA isolated from control (LMP) or miHO1 infected (miHO-1) cells was labeled and hybridized to human genome-wide gene level 1.0 ST arrays. Among 214 differentially expressed genes with statistical significance (adjusted p-value < 0.05), 67 genes were expressed at higher levels in HO-1 expressing control (LMP) cells and 147 genes in cells deficient in HO-1 (miHO-1, see Additional file 1). Top 30 differentially expressed genes are shown in Table 1. An obvious feature of HO-1 was its effect on the expression of genes which are either directly or indirectly linked to cell adhesion and the integrity or remodelling of extracellular matrix (CD24, HOOK1, LAYN, HEY1, MME, RRAS2, FZD3, KIF14, KIF18A, DAAM1, BCL6, PLS1, ERBB2IP, BGN, FSD1, IFI30, LGALS3BP, FMNL1, TMSL3, CORO1A, TFF1, CLEC11A, ADAM8, ECM1, PLTP, TGFB1, PXDN, COL21A1 and MMP2; Table 1, and Additional Files 1 &2).

**Table 1:** Top 30 genes up- or downregulated statistically significant more than 2-fold in BeWo control (LMP) cells compared with cells deficient in HO-1 (miHO-1)

Probe Set ID	Mean Fold Change (LMP vs miHO-1)	Adj. P-Value	Gene Symbol	Gene Name
**Upregulated in HO-1 expressing control (LMP) cells**:				
NM_005330_at	7,82	0,0073	HBE1	hemoglobin, epsilon 1
NM_024913_at	7,74	0,0067	C7orf58	chromosome 7 open reading frame 58
NM_001105533_at	5,56	0,0073	C7orf58	chromosome 7 open reading frame 58
NM_206828_at	5,10	0,0077	NLRP7	NLR family, pyrin domain containing 7
NM_002133_at	5,09	0,0262	HMOX1	heme oxygenase (decycling) 1
NM_139176_at	4,90	0,0081	NLRP7	NLR family, pyrin domain containing 7
NM_004530_at	4,83	0,0098	MMP2	matrix metallopeptidase 2 (gelatinase A)
XM_001720850_at	4,48	0,0092	LOC100134134	similar to peroxidasin homolog
NM_030820_at	4,34	0,0223	COL21A1	collagen, type XXI, alpha 1
NM_012293_at	4,27	0,0092	PXDN	peroxidasin homolog (Drosophila)
XM_001715515_at	3,86	0,0106	LOC100131375	similar to peroxidasin homolog
NM_000660_at	3,60	0,0306	TGFB1	transforming growth factor, beta 1
NM_001312_at	3,39	0,0146	CRIP2	cysteine-rich protein 2
NM_004425_at	2,99	0,0262	ECM1	extracellular matrix protein 1
NM_006227_at	2,91	0,0306	PLTP	phospholipid transfer protein
NM_182676_at	2,91	0,0306	PLTP	phospholipid transfer protein
XM_001718318_at	2,91	0,0318	LOC100131307	hypothetical protein LOC100131307
NM_000413_at	2,88	0,0262	HSD17B1	hydroxysteroid (17-beta) dehydrogenase 1
NM_178422_at	2,87	0,0301	PAQR7	progestin and adipoQ receptor family member VII
NM_022664_at	2,86	0,0262	ECM1	extracellular matrix protein 1
NM_001109_at	2,76	0,0284	ADAM8	ADAM metallopeptidase domain 8
NM_000391_at	2,64	0,0306	TPP1	tripeptidyl peptidase I
XM_001726123_at	2,64	0,0262	LOC100133842	similar to lectin, galactoside-binding, soluble, 3 binding protein
NM_003045_at	2,63	0,0262	SLC7A1	solute carrier family 7, member 1
NM_005931_at	2,63	0,0231	MICB	MHC class I polypeptide-related sequence B
NM_000041_at	2,61	0,0262	APOE	apolipoprotein E
NM_006927_at	2,60	0,0262	ST3GAL2	ST3 beta-galactoside alpha-2,3-sialyltransferase 2
NM_001042423_at	2,60	0,0334	SLC16A3	solute carrier family 16, member 3 (monocarboxylic acid transporter 4)
NM_004207_at	2,59	0,0332	SLC16A3	solute carrier family 16, member 3 (monocarboxylic acid transporter 4)
**downregulated in HO-1 expressing control (LMP) cells**:				
NM_013230_at	-22,27	0,0067	CD24	CD24 molecule
XM_001725629_at	-19,19	0,0067	CD24	CD24 molecule
NM_003810_at	-14,22	0,0067	TNFSF10	tumor necrosis factor (ligand) superfamily, member 10
NM_032812_at	-6,27	0,0072	PLXDC2	plexin domain containing 2
XM_001726122_at	-3,66	0,0206	FLJ32810	hypothetical protein FLJ32810
NM_006982_at	-3,37	0,0155	ALX1	ALX homeobox 1
NM_015888_at	-3,34	0,0262	HOOK1	hook homolog 1 (Drosophila)
NM_178834_at	-3,34	0,0315	LAYN	layilin
NM_003063_at	-3,27	0,0262	SLN	sarcolipin
NM_030972_at	-3,25	0,0178	ZNF611	zinc finger protein 611
NM_032021_at	-3,15	0,0178	TMEM133	transmembrane protein 133
NM_005028_at	-3,14	0,0206	PIP4K2A	phosphatidylinositol-5-phosphate 4-kinase, type II, alpha
XM_001715384_at	-3,09	0,0256	LOC100134369	similar to golgi phosphoprotein 2
NM_017423_at	-3,06	0,0262	GALNT7	N-acetylgalactosaminyltransferase 7
NM_001008401_at	-3,00	0,0306	ZNF761	zinc finger protein 761
XM_001127597_at	-2,98	0,0178	FLJ32810	hypothetical protein FLJ32810
NM_030925_at	-2,95	0,0178	CAB39L	calcium binding protein 39-like
NM_001079670_at	-2,92	0,0178	CAB39L	calcium binding protein 39-like
NM_001726_at	-2,88	0,0297	BRDT	bromodomain, testis-specific
NM_177937_at	-2,87	0,0178	GOLM1	golgi membrane protein 1
NM_014827_at	-2,81	0,0321	ZC3H11A	zinc finger CCCH-type containing 11A
NM_001042482_at	-2,78	0,0306	TPK1	thiamin pyrophosphokinase 1
NM_004052_at	-2,77	0,0306	BNIP3	BCL2/adenovirus E1B 19 kDa interacting protein 3
NM_006424_at	-2,75	0,0262	SLC34A2	solute carrier family 34 (sodium phosphate), member 2
NM_005900_at	-2,73	0,0262	SMAD1	SMAD family member 1
NM_001008397_at	-2,72	0,0262	GPX8	glutathione peroxidase 8
NM_018169_at	-2,71	0,0284	C12orf35	chromosome 12 open reading frame 35
NR_003942_at	-2,68	0,0318	SNORD76	small nucleolar RNA, C/D box 76
XM_001726844_at	-2,66	0,0375	LOC100130123	PRO2870
NM_207189_at	-2,65	0,0276	BRDT	bromodomain, testis-specific

Mean fold change values of n = 2 experiments.

### Pathway prediction analyses

To further explore the dataset, GSEA [20] was used to identify groups of functionally related genes with expression patterns that correlate with HO-1 expression. GSEA is a method for interpreting gene expression data that focus on groups of genes sharing common biological function, chromosomal location or regulation. This approach can show important effects on pathways, which might be missed in single-gene analyses [20]. Fig. 1C displays the top 10 pathways regulated by HO-1 expression in BeWo cells. Amongst others, HO-1 expressing BeWo cells were significantly enriched in pathways regulating extracellular matrix orchestration and signal transduction. Plotting of the enrichment score vs the rank-ordered gene list for the top-scoring gene set Éxtracellular region' illustrates increased expression of ECM molecules and their remodeling enzymes in HO-1 expressing BeWo LMP cells (Fig. 1D). A more detailed analysis of this pathway revealed enhanced expression of several ECM molecules including extracellular matrix-1 (ECM1), collagen type IX, α3 (COL9A3, sarcoglycans β and -ε (SGCβ and SBCε), and the matrix remodeling factors MMP2 and TGFβ1 (Fig. 1E). The significant gene sets 'receptor binding', integral to plasma membrane' and 'system development' contained further genes related to cell plasticity and ECM organization, including IGF2, placental growth factor (PGF), collagen type I α1 (COL1A1), fibroblast growth factor receptors 3 and 4 (FGFR-3,-4), ADAM8, (see Additional File 3). These observations suggest that HO-1 expressing cells produce factors relevant to cell-matrix adhesion as well as their degrading enzymes.

### HO-1 gene signature in 190 human tumors

**Figure 2:**
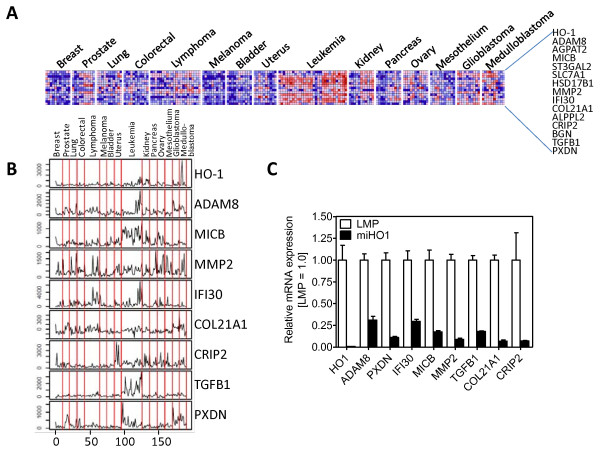
**HO-1 target gene expression in 190 human tumors**. (A) The heatmap shows the expression pattern of 14 HO-1 target genes including HO-1 itself in the 190 human tumors of the 14 most common cancer types included in the GCM database. Red and blue squares denote high versus low levels of expression of each individual gene normalized to its mean expression across all tumor samples, respectively. (B) Relative gene expression of 8 selected HO-1 target genes. The Y-axis displays the normalized DNA Microarray signal across 190 tumor samples (comprising the X-axis). Cancer types are shown above the plot. (C) mRNA levels were measured by qRT-PCR using the RNA samples isolated from HO-1 expressing (LMP) and HO-1 silenced (miHO-1) BeWo cells. The expression values were normalized relative to Arp. The levels of mRNA in LMP and miHO-1 cells are shown in percentage relative to LMP cells (set to 100%). Bars represent mean (+/- SEM) of three independent experiments.

**Figure 3:**
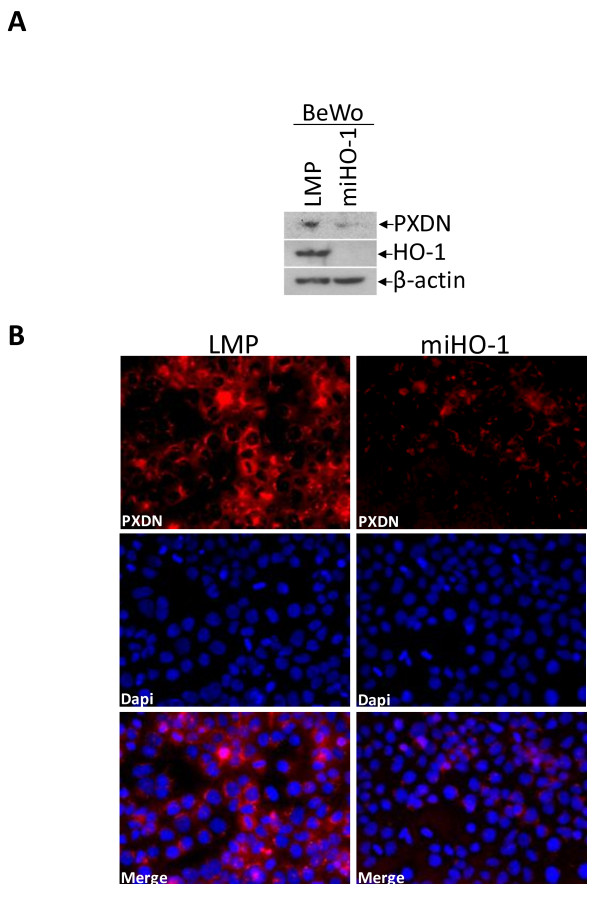
**Knock-down of HO-1 in BeWo cells diminishes PXDN protein expression**. (A) PXDN detection by western blotting of LMP and miHO-1 BeWo cells. β-actin indicates equal loading. A representative example is shown. (B) Immunofluorescent detection of PXDN (=red) and nuclei (DAPI = blue) in cultured LMP and miHO-1 BeWo cells. Fields shown are representative of each population.

We next determined whether expression of the putative HO-1 target genes identified in BeWo cells (Additional File 1) correlates with HO-1 expression levels in human tumors. For these purpose, we performed data mining using the GCM database. This database includes the expression profiling data of 16,063 genes of 190 individual tumors of the 14 human cancer types. Using R/Bioconductor, we ranked 7978 genes (filtering described in methods) according to their level of coexpression with HO-1. The Pearson distance was used as an unbiased measure of the expression pattern similarity of the target gene with the expression pattern of HO-1. Using the data of Additional File 1, we then selected the top 14 individual genes, expression of which most uniformly correlated with that of HO-1 both in BeWo LMP cells and in 190 human tumors (Fig. 2A and Table 2). To confirm that the coexpression of these 14 genes with HO-1 is statistically significant we applied KS statistics. One hundred thousand trials with a randomly selected set of 14 genes undermined the high statistical significance of the 14 identified genes (P = 0.00002). Fig. 2B shows the expression pattern of the 14 highly significant HO-1 target genes in the 190 tumor samples, which include ADAM8, AGPAT2, MICB, ST3GAL2, SLC7A1, HSD17B1, MMP2, IFI30, COL21A1, ALPPL2, CRIP2, BGN, TGFB1 and PXDN. To corroborate our results, we used qRT-PCR to determine the mRNA levels of 8 HO-1 target genes in BeWo LMP and miHO-1 cells (Fig. 2C). These 8 HO-1 target genes were selected based on their putative role in regulation of cell plasticity/motility based on Gene Ontology classification and PubMed searches. According to our results, the expression levels of these genes were the lowest in miHO-1 cells. Western blotting of LMP and miHO-1 BeWo cell extracts confirmed the increased levels of PXDN in LMP cells (Fig. 3A). Immunofluorescence analysis of routinely cultured, subconfluent LMP and miHO-1 BeWo cells further corroborated western blotting data, showing increased (mostly perinuclear) PXDN staining in LMP cells (Fig. 3B).

**Table 2:** HO-1 target genes

Gene Accession	Gene Symbol	Distance from HO-1	Fold Change (LMP/miHO-1)
NM_001109	ADAM8	0.5570885	2,76
NM_001012727	AGPAT2	0.6074327	2,05
NM_005931	MICB	0.66872585	2,63
NM_009179	ST3GAL2	0.680627	2,60
NM_003045	SLC7A1	0.70778143	2,63
NM_000413	HSD17B1	0.7225144	2,88
NM_008610	MMP2	0.7414118	4,83
NM_006332	IFI30	0.7581253	2,24
NM_030820	COL21A1	0.7659843	4,34
NM_031313	ALPPL2	0.77093726	2,10
NM_001312	CRIP2	0.78032595	3,39
NM_001711	BGN	0.8124546	2,01
NM_000660	TGFB1	0.82223743	3,60
NM_012293	PXDN	0.82782316	4,27

NOTE: Distance from HO-1 corresponds to ranking derived from the Pearson distance from HO-1. Fold change (LMP/miHO-1), the ratio of gene expression levels in HO-1 expressing cells versus HO-1 silenced cells.

### HO-1 and PXDN colocalize in invasive trophoblast

**Figure 4:**
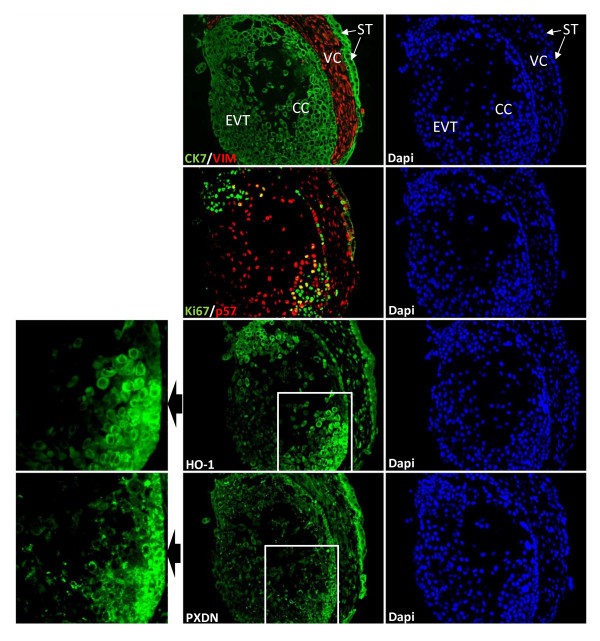
**PXDN and HO-1 co-localize in first trimester placenta**. Immunohistochemical analyses of serial sections of placental tissues (between 7 and 14th weeks of gestation) stained with different antibodies, counterstained with DAPI and analysed by fluorescence microscopy as described in Materials and Methods. Trophoblast and stromal cells are marked by Cytokeratin 7 (CK7, green) and Vimentin (VIM, red) staining, respectively. Ki67 (red) staining indicates poorly invasive but proliferative EVT, whereas p57 (green) positive cells account for invasive, differentiated EVT. HO-1 and PXDN were stained on separate serial sections. Photographs were taken at 400-fold magnification. VC, villous core; EVT, extravillous trophoblast; CC, cell column; ST, syncytiotrophoblast. Note the relative higher abundance of PXDN and HO-1 in CTB as compared with EVT. Magnification: 200x.

To confirm a link of HO-1 with PXDN, we determined the expression of HO-1 and PXDN in first trimester placenta tissues. Among the Cytokeratin-positive (=villous, extravillous as well a ssyncytiotrophoblast) cells, Ki67- or p57-staining indicated proliferating (non-invasive) or invasive, differentiated extravillous trophoblast cells, respectively (Fig. 4). Immunofluorescence analysis of serial sections revealed pan-trophoblastic HO-1 and PXDN staining (Fig. 4), however, proximal extravillous trophoblasts in the cell column stained strongest for HO-1 and PXDN. Based on our immunostaining data, we concluded that the expression of HO-1 is coupled to an up-regulation of PXDN in first trimester placenta.

### HO-1 affects cell adhesion to extracellular matrix molecules via PXDN

**Figure 5:**
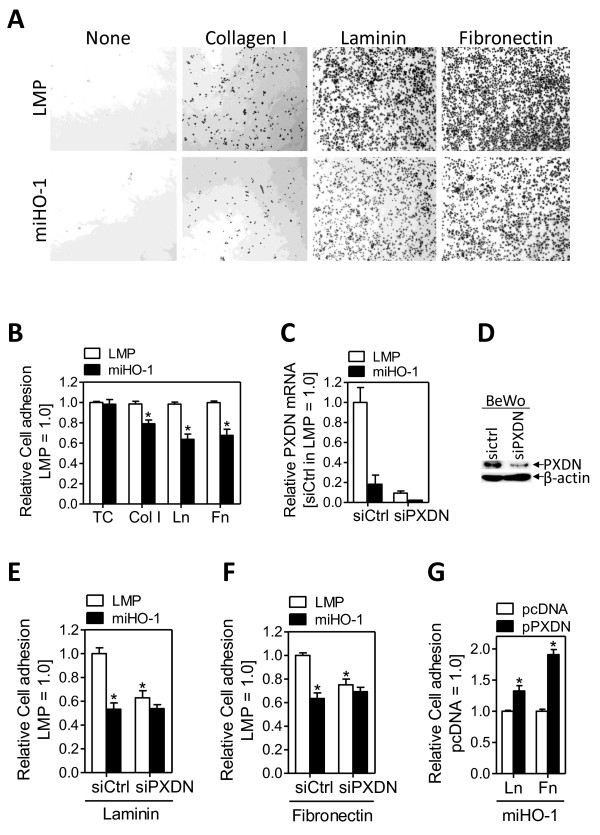
**HO-1 mediates cell adhesion via PXDN in BeWo choriocarcinoma cells via PXDN**. (A) Control-infected (LMP) or miHO-1 (miHO-1) infected BeWo cells were seeded on fibronectin (FN), collagen type I (Col I), laminin (Ln) or tissue culture plastic (TC) and assayed for adhesion as described in *Materials&Methods*. Photographs were taken at a 40x magnification. (B) The absorbance of fixed and crystal-violet stained cells at 550 nm of ctrl (LMP) infected cells were arbitrarily set to 100% in each experiment. Note that HO-1 deficient BeWo cells (miHO-1) became less adherent than LMP cells expressing HO-1 endogenously (*P < 0.05 vs LMP cells). PXDN knocked down in BeWo LMP and miHO-1 cells after transient transfection with a control (siCtrl) or PXDN-specific (siPXDN) siRNA, as determined by real-time PCR (C) and western blotting (D). β-actin indicates equal loading. Effect of PXDN-knockdown on cell adhesion to fibronectin (E) or laminin (F) in control-infected (LMP) or miHO-1 (miHO-1) infected BeWo cells. For comparison, OD-values of ctrl (LMP) infected cells were arbitrarily set to 100% in each experiment. Note that HO-1 expressing cells (LMP), but not HO-1 deficient cells (miHO-1) became less adherent following PXDN-knockdown (*P < 0.05 vs siCtrl-treated LMP cells). (G) Effect of ectopic PXDN expression on cell adhesion of miHO1 BeWo cells. For comparison, OD-values of cells transfected with an empty control plasmid (pCDNA) were arbitrarily set to 100% in each experiment. Note that PXDN overexpressing cells (pPXDN), but not mock-transfected cells (pcDNA) became more adherent (*P < 0.05 vs pcDNA treatment).

We examined the effect of HO-1 knockdown on the attachment of BeWo LMP and miHO-1 cells to fibronectin, laminin and collagen type I using cell adhesion assays. In this assay, nonadherent cells were removed gently and the remaining adherent cells were fixed, stained and analysed by light microscopy. As shown in Fig. 5A, HO-1 expressing cells (LMP) became much more adherent compared with HO-1 deficient (miHO1) cells. The adherent cells were measured at 550 nm following staining with crystal violet. As shown in Fig. 5B, the absorbance of LMP cells was significantly higher than that of miHO-1cells (P > 0.05). This effect was more pronounced in the order Laminin > Fibronectin > Collagen type I. It is noteworthy that very few cells adhered to control wells (termed 'Ctrl'). To examine if the HO-1 target gene PXDN is accountable for the increased adhesivenss of HO-1 expressing BeWo cells, we repeated adhesion assays with BeWo cells silenced for PXDN expression. We observed diminished PXDN mRNA and protein levels two days after transfection of LMP and miHO-1 BeWo cells with a PXDN-specific siRNA, but not with a negative control siRNA, (Fig. 5C and 5D, respectively). We evaluated effects of PXDN knockdown on cell adhesion to Fibronectin and Laminin, as BeWo cells most efficiently adhere to these matrix proteins. Transfection with a control siRNA did not alter the inhibitory effect of reduced HO-1 levels on adhesion of BeWo cells to Fibronectin or Laminin (Fig. 5E and 5F). While PXDN-knockdown did not alter cell adhesion properties of HO-1 deficient BeWo cells (miHO-1), siRNA-mediated PXDN-knockdown abolished the stimulatory effect of HO-1 on cell adhesion observed in LMP cells (Fig. 5E and 5F). To minimize the risk of off-target effects, we repeated the cell adhesion experiments with an alternative siRNA against PXDN with similar results (Additional File 4). To undermine a role of PXDN in cell adhesion, we transiently overexpressed PXDN in BeWo miHO1 cells. Ectopic expression of PXDN (pPXDN) resulted in enhanced adhesion to Laminin and Fibronectin, as compare to cells transfected with a control pasmid (Fig. 5G).

**Figure 6:**
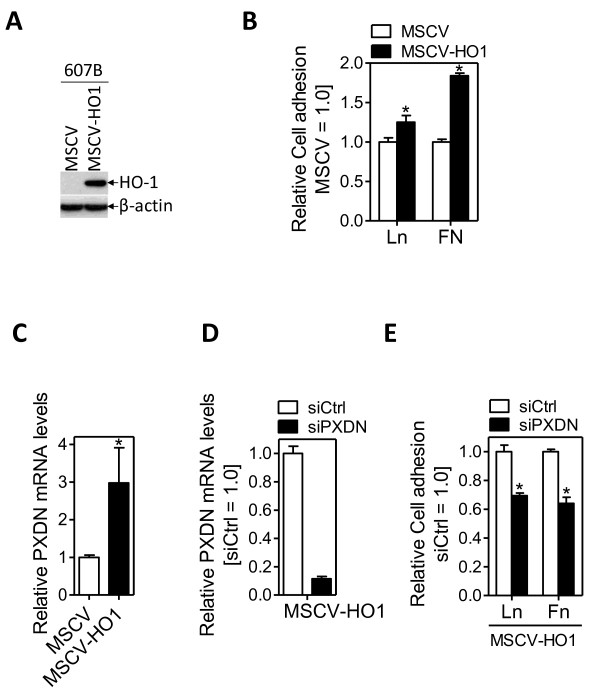
**HO-1 mediates cell adhesion via PXDN in 607B melanoma cells**. (A) Western blot analysis of ctrl (MSCV) or cells transduced with a HO-1 cDNA (MSCV-HO1) demonstrates efficient HO-1 overexpression in 607B melanoma cells. β-actin indicates equal loading. A representative example is shown. (B) Control-infected (MSCV) or HO-1 transduced (MSCV-HO-1) 607B cells were seeded on Fibronectin (FN), Laminin (Ln) or tissue culture plastic and assayed for adhesion as described in *Materials&Methods*. Note that HO-1 overexpressing 607B cells (MSCV-HO1) became more adherent than MSCV cells (*P < 0.05 vs MSCV cells). (C) PXDN mRNA levels were measured by qRT-PCR using the RNA samples isolated from control (MSCV) or HO-1 overexpressing (MSCV-HO1) 607B cells. The expression values were normalized relative to Arp. The levels of PXDN mRNA in MSCV and MSCV-HO1 cells are shown in percentage relative to MSCV cells (set to 100%). Bars represent mean (+/- SEM) of three independent experiments. (D) PXDN mRNA knocked down in 607B MSCV-HO1 cells after transient transfection with a control (siCtrl) or PXDN-specific (siPXDN) siRNA, as determined by real-time PCR. (E) Effect of PXDN-knockdown on cell adhesion to fibronectin or laminin in HO-1 overexpressing 607B cells. For comparison, OD-values of siCtrl infected cells were arbitrarily set to 100% in each experiment. Note that PXDN-silenced cells (siPXDN), but not siCtrl-treated (siCtrl) cells became less adherent following PXDN-knockdown (*P < 0.05 vs siCtrl-treated MSCV-HO1 cells).

**Figure 7:**
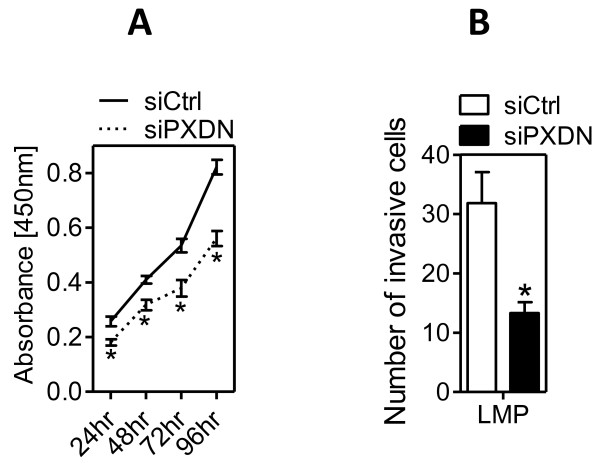
**PXDN-knockdown inhibits BeWo cell invasion and cell growth**. (A). Invasion assay in matrigel-coated Boyden chambers of BeWo cells with knockdown of PXDN. Invaded cells were counted by photographing the membrane through the microscope. Bar graphs represent mean cell number +/- SEM of three independent experiments. *p < 0.05 vs siCtrl-treated BeWo-LMP cells. (B) The cell growth of siCtrl and siPXDN-treated BeWo LMP cells were examined by BrdU assay over a 4-day period. Error bars indicate SEM (*P < 0.05 vs siCtrl-treated BeWo-LMP cells).

To verify that the effects of HO-1 on cell adhesion and PXDN expression are truly related to HO-1, we generated a HO-1 gain-of-function cell model using 607B melanoma cells, which have no detectable endogenous HO-1 expression. As shown by western blotting (Fig. 6A), retroviral HO-1 gene transfer into 607B cells resulted in stable HO-1 overexpression ('MSCV-HO1') as compared to cells transduced with a virus containing empty retroviral backbone ('MSCV'). Adhesion to Fibronectin and Laminin was more pronounced in 607B cells overexpressing HO-1 (MSCV-HO1) as compared to control infected cells (MSCV; Fig. 6B). Furthermore, MSCV-HO1 cells expressed higher levels of PXDN, compared with MSCV control cells (Fig. 6C). To investigate if PXDN has pro-adhesive properties in 607B cells, similar to BeWo cells, adhesion assays were repeated using PXDN-silenced MSCV-HO1 cells. siPXDN, but not siCtrl-treatment of 607B MSCV-HO1 cells efficiently knocked-down PXDN mRNA levels (~10-fold reduction; Fig. 7D). Furthermore, PXDN-knockdown in 607B MSCV-HO1 cells resulted in a significant reduction in cell adhesion to Fibronectin and Laminin, as compared to siCtrl-treated cells (Fig. 6E). Subsequently, we examined the effect of silenced PXDN expression on BeWo cell growth and invasion. Compared to siCtrl transfected cells, PXDN-silenced cells showed significant decreased cell growth over 96 hrs (Fig. 7A). Of note, knockdown of HO-1 in BeWo cells did not affect cell proliferation (data not shown). When testing the abilities of BeWo cells to invade through the 8-μm pores on the polycarbonate membrane coated with matrigel, we found that the knock-down of endogenous PXDN expression significantly reduced cell invasion as compared to siCtrl treated BeWo LMP cells (P < 0.05; Fig. 7B)

## Discussion

In cancer, HO-1 influences tumor cell survival, apoptosis, invasion and metastasis as well as resistance of certain tumors to chemotherapeutic agents [7,17]. These changes suggest alterations of signal transduction and transcription pathways, which HO-1 affects either directly or indirectly. To identify these regulatory mechanisms and to determine the identity of the universal genes, expression of which is affected by HO-1, we silenced HO-1 expression in BeWo choriocarcinoma cells ('miHO-1') and performed gene expression profiling of these cells relative to BeWo cells which express HO-1 endogenously ('LMP'). An interesting aspect of the 214 identified genes whose expression was affected by HO-1, was the regulation of multiple genes linked to cell plasticity/motility and ECM maintenance. In the course of invasion tumor cells leave normal structures by passing through basal membrane and migrate into the surrounding stroma. These events include significant changes in cell morphology as well as close interaction of cells with extracellular matrix (ECM) and structural rearrangement of the latter. Further evidence for a role of HO-1 in modulating cell plasticity was revealed by pathway prediction analysis, which demonstrated modulation of genes of the extracellular region as well as underlying signal transduction pathways (GSEA; Fig 1). Consistent with our data, TGFB1 was identified as a HO-1 target gene in a microarray comparison of prostate cancer cells with varying HO-1 protein levels [12].). Several potential mechanisms underlying gene regulation by HO-1 can be envisioned that also emphasize a potential role of the enzymatic products of HO-1: regulation of signaling pathways including ERK and p38 MAPK [13], Akt/Protein kinase B [5], and transcription factors such as AP-1, AP-2, Brn-3 [29], PPARγ [25], NF-kappaB [30], HSF-1 [31] and HIF1α [32]. Heme containing (and carbon monoxide) responsive transcription factors such as NPAS2 [33] and REV-ERBα/REV-ERBβ [34,35] modulate gene expression in response to the HO-1 enzymatic product carbon monoxide. Recent studies revealed the nuclear localization of HO-1, pointing to its role as a potential transcription factor or coregulator [29,36] Of note, we detected a fraction of total cellular HO-1 protein in the nucleus of BeWo cells (data not shown). Further studies are warranted to investigate potential signaling pathways triggered by HO-1, (including the role of nuclear HO-1) in gene regulation.

To provide unbiased proof for the role of HO-1 in genome-wide transcriptional regulation, irrespective of the cancer tissue type, we performed a metaprofiling analysis using the GCM database of 190 human tumors of 14 different types. The motivation of this data mining strategy was to identify which genes from the 214 putative HO-1 target genes, determined in BeWo cells, most closely correlated with the expression of HO-1 in 190 tumor samples. This unbiased comparative analysis revealed 14 HO-1 universal target genes: proteolytic ADAM8 and MMP2, acyltransferase AGPAT2, cell surface protein MICB, extracellular glycosylase ST3GAL2, amino acid transporter SLC7A1, steroid dehydrogenase HSD17B1, thiol reductase IFI30, alkaline phosphatase ALPPLA2, intracellular adapter protein CRIP2, exracellular matrix constituents BGN and COL21A1, multifunctional cytokine TGFB1, and peroxidase PXDN. The expression of these genes is strongly correlated with that of HO-1 (P = 0.00002). The results of our data mining and our subsequent statistical analyses were validated by using qRT-PCR, Western blotting, and immunostaining of LMP and miHO1 cells. Immunofluorescence staining of first trimester placenta specimens confirmed that HO-1 immunoreactivity is coupled to that of PXDN in trophoblast cells (Fig. 4), which share the capacity to migrate and invade surrounding tissues similar to malignant cells [37]. Based on these results, we suggest that HO-1 stimulates multiple transcriptional changes and affects several cellular pathways, including extracellular matrix organization (MMP2, ADAM8, TGFβ1, BGN, COL21A1, PXDN), signaling (CRIP2, MICB), amino acid transport and glycosylation (SLC7A1 and ST3GAL2), estrogen and phospholipid biosynthesis (AGPAT2 and HSD17B1), protein stabilization (IFI30) and phosphorylation (ALPPL2). Many of these genes are directly associated with cancer; further studies are warranted to identify the role of the HO-1 associated genes in the tumorigenic proteries of HO-1.

Given that cell adhesion is intrically linked to tumor progression/invasion, and that the HO-1 gene signature features many regulators of cell adhesion, we investigated potential effects of HO-1 on cell adhesion in HO-1 silenced BeWo cells and HO-1 overexpressing 607B melanoma cells. Knockdown of HO-1 in BeWo cells reduced adhesion to various ECM molecules, having strongest effect on Laminin (Fig. 5). Stronger adhesion of 607B cells overexpressing HO-1 confirmed a positive role of HO-1 in cell adhesion (Fig. 7). Previously, we have shown that loss of HO-1 expression in BeWo cells resulted in increased cell motility, based on boyden chamber assays [25]. Thus, at least in BeWo cells, knockdown of HO-1 decreases cell adhesion with a concomitant increase in cell motility. A reduction in cell adhesion with a concomitant increase in cell motility is one hallmark of mesenchymal-amoeboid transition (MAT), a process describing a change in (cancer cell) movement from mesenchymal to amoeboid mode. Such type of movement was shown to be characteristic of certain malignancies, including prostate cancer [10,11].

We hypothesized that one of the HO-1 signature genes, many of which represent potential regulators of cell plasticity, mediates the adhesion-promoting effect of HO-1. One promising and novel candidate was PXDN, which could alter cell-ECM interaction either by stabilization of the ECM through protein-protein interactions via leucine-rich repeats and immunoglobulin loops, as well as by enzymatically formed tyrosine-tyrosine crosslinks [38]. PXDN, also known as MG50, is a peroxidase associated with the endoplasmatic reticulum, and expressed in melanoma, breast cancer, colon cancer, ovarian cancer, renal carcinoma as well as metastatic gliomas [4,38-40] Silencing of PXDN abolished the adhesion-promoting effect of endogenous HO-1 in BeWo (LMP) and 607B (MSCV-HO1) cells (Fig. 5 and Fig. 6), while PXDN knockdown did not affect cell adhesion in HO-1 deficient cells. We hypothesize that the PXDN dosage may be very critical for the adhesive response, as PXDN levels in miHO-1 cells treated with a PXDN specific siRNA were ~50 times lower compared to LMP cells (Fig. 5): If inhibition of BeWo cell adhesion correlates with PXDN - levels, maybe there exists a threshold level for PXDN. However, the phenotype of miHO1 cells could be rescued by PXDN overexpression (Fig. 6G). The reduced (~50%) matrigel invasion of PXDN-silended BeWo cells is most likely due to pro-proliferative properties of PXDN (Fig 7). However, additional mechanisms must prevail as cell growth in PXDN silenced cells was inhibited by approximately 30% after 24 hrs, the duration of the cell invasion assay. Importantly, to our knowledge, this is the first time showing functional effects of PXDN expression levels on cell adhesion and invasion. Further extensive experiments are needed to determine the molecular mechanism by which PXDN modulates cell adhesion and invasion, and how it is linked to the adhesion-promoting properties of HO-1.

To conclude, our unbiased large scale genome-wide studies clarified, for the first time, the molecular signature of HO-1 in cancer and identified the genes which are functionally, universally, and most consistently linked with HO-1 expression among multiple tumor types. The identification of the HO-1 target genes will undoubtedly help to understand the complex network of cellular and molecular events, which are linked to the role of HO-1 in cancer. Ongoing studies will shed light on the functional significance of these individual genes.

## Competing interests

The authors declare that they have no competing interests.

## Authors' contributions

ST carried out the GeneChip and bioinformatic as well as statistical analysis and drafted the manuscript. AJ carried out adhesion assays and western blotting. SH and MK carried out the immunostaining. MM designed primers and performed real-time PCR measurements. JH generated retroviral constructs, conducted retroviral gene transductions and cell proliferation assays. JL performed cell invasion assays and transient transfections. HP and OW participated in the design and coordination of the study. MB conceived of the study, and participated in its design and coordination and helped to draft the manuscript. All authors read and approved the final manuscript.

## Supplementary Material

Additional file 1 **Transcriptional signature of HO-1 in BeWo choriocarcinoma cells**. The data provided represent the 214 differentially expressed genes with statistical significance (adjusted p-value < 0.05) between HO-1 expressing control ('LMP') cells and cells deficient in HO-1 ('miHO-1'). Click here for file

Additional file 2 **quantitative real-time PCR validation of 5 genes regulated upon HO-1 knockdown**. Graphical presentation for five differentially expressed genes selected for qRT-PCR validation. mRNA levels were measured by qRT-PCR using the RNA samples isolated from HO-1 expressing (LMP) and HO-1 silenced (miHO-1) BeWo cells. The expression values were normalized relative to Arp. The levels of mRNA in LMP and miHO-1 cells are shown in percentage relative to LMP cells (set to 100%). Bars represent mean (+/- SEM) of three independent experiments.Real-time PCR verification of genes statistically significant overexpressed in cells deficient of HO-1. Primers for selected genes were designed using Primer3 software http://frodo.wi.mit.edu/cgi-bin/primer3/primer3_www.cgi with the following sequences: TNFSF10 (CTGGGACCAGAGGAAGAAGC, fwd; GCTCAGGAATGAATGCCCAC, rev), PEG3 (TCCTCACCACCTCACTCAGTC, fwd; GGTCTCGTGGCTCCATGTC, rev), GOLM1 (AGCGTGGACCTCCAGACAC, fwd; CTGCGGACCCTGCCTTCC, rev), CAB39L (CCAACAGAAGCAGTGGCTCA, fwd; GCTGCAGGTCAGCTATCAGTG, rev), CD24 (CCAACTAATGCCACCACCAAG , fwd; TGTTGACTGCAGGGCACCAC, rev). The RNA-amount of the human Arp gene was used as an internal control. Data were analyzed according to the 2^-ΔΔCT ^method [24]. Click here for file

Additional file 3 **Pathway analysis in HO-1 deficient BeWo cells**. Graphical presentation of 9 gene sets as heatmaps that correlate with HO-1 expression in BeWo cells, identified by GSEA. Downregulated genes are shown in blue and upregulated genes in red. Click here for file

Additional file 4 **PXDN-knockdown in BeWo cells using an alternative siRNA oligo targeting human PXDN**. Upper panel: PXDN knocked down in BeWo LMP cells after transient transfection with a control (siCtrl) or PXDN-specific (siPXDN#2) siRNA (Invitrogen, oligo ID: HSS187891) , as determined by real-time PCR. Lower panel: Effect of PXDN-knockdown using siPXDN#2 on cell adhesion to fibronectin or laminin in control-infected (LMP) BeWo cells. For comparison, OD-values of LMP cells treated with a control siRNA (siCtrl) were arbitrarily set to 100% in each experiment. Note that HO-1 expressing cells (LMP) became less adherent following PXDN-knockdown. Click here for file
