# Supplementary figures and images for: Transcriptome analysis of human cancer reveals a functional role of Heme Oxygenase-1 in tumor cell adhesion
Source: Mol Cancer. 2010 Jul 28;9:200. doi: 10.1186/1476-4598-9-200 (PMC2917430; doi:10.1186/1476-4598-9-200)

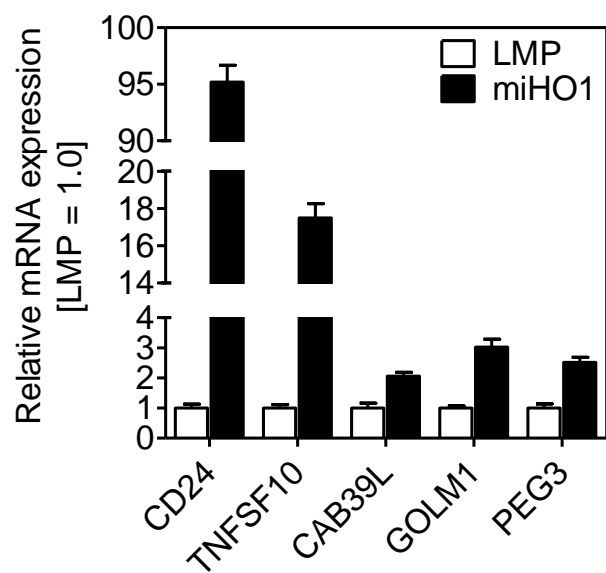

Supplement: Additional file 2 — quantitative real-time PCR validation of 5 genes regulated upon HO-1 knockdown. Graphical presentation for five differentially expressed genes selected for qRT-PCR validation. mRNA levels were measured by qRT-PCR using the RNA samples isolated from HO-1 expressing (LMP) and HO-1 silenced (miHO-1) BeWo cells. The expression values were normalized relative to Arp. The levels of mRNA in LMP and miHO-1 cells are shown in percentage relative to LMP cells (set to 100%). Bars represent mean (+/- SEM) of three independent experiments.Real-time PCR verification of genes statistically significant overexpressed in cells deficient of HO-1. Primers for selected genes were designed using Primer3 software http://frodo.wi.mit.edu/cgi-bin/primer3/primer3_www.cgi with the following sequences: TNFSF10 (CTGGGACCAGAGGAAGAAGC, fwd; GCTCAGGAATGAATGCCCAC, rev), PEG3 (TCCTCACCACCTCACTCAGTC, fwd; GGTCTCGTGGCTCCATGTC, rev), GOLM1 (AGCGTGGACCTCCAGACAC, fwd; CTGCGGACCCTGCCTTCC, rev), CAB39L (CCAACAGAAGCAGTGGCTCA, fwd; GCTGCAGGTCAGCTATCAGTG, rev), CD24 (CCAACTAATGCCACCACCAAG , fwd; TGTTGACTGCAGGGCACCAC, rev). The RNA-amount of the human Arp gene was used as an internal control. Data were analyzed according to the 2-ΔΔCT method [24]. [file 1476-4598-9-200-S2.PDF]

Additional File 4

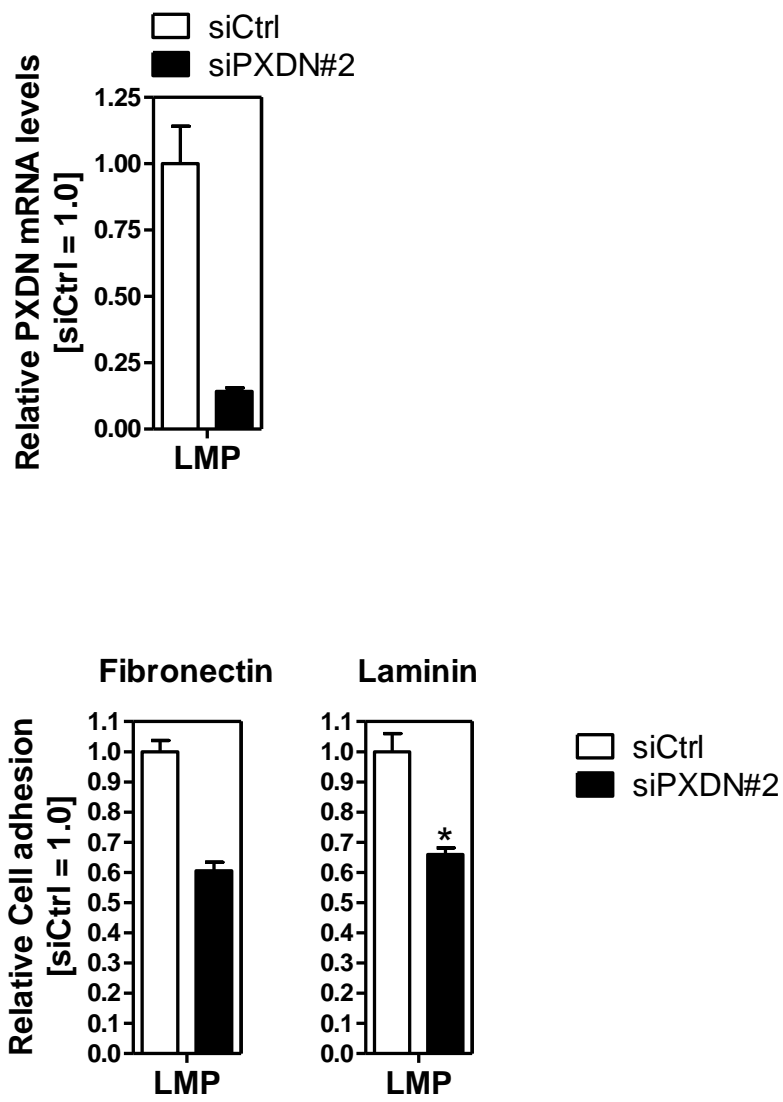

Supplement: Additional file 4 — PXDN-knockdown in BeWo cells using an alternative siRNA oligo targeting human PXDN. Upper panel: PXDN knocked down in BeWo LMP cells after transient transfection with a control (siCtrl) or PXDN-specific (siPXDN#2) siRNA (Invitrogen, oligo ID: HSS187891) , as determined by real-time PCR. Lower panel: Effect of PXDN-knockdown using siPXDN#2 on cell adhesion to fibronectin or laminin in control-infected (LMP) BeWo cells. For comparison, OD-values of LMP cells treated with a control siRNA (siCtrl) were arbitrarily set to 100% in each experiment. Note that HO-1 expressing cells (LMP) became less adherent following PXDN-knockdown. [file 1476-4598-9-200-S4.PDF]
