# Supplementary material for: Transcriptome analysis of human cancer reveals a functional role of Heme Oxygenase-1 in tumor cell adhesion
Source: Mol Cancer. 2010 Jul 28;9:200. doi: 10.1186/1476-4598-9-200 (PMC2917430; doi:10.1186/1476-4598-9-200)
Supplement: Additional file 3 — Pathway analysis in HO-1 deficient BeWo cells. Graphical presentation of 9 gene sets as heatmaps that correlate with HO-1 expression in BeWo cells, identified by GSEA. Downregulated genes are shown in blue and upregulated genes in red. [file 1476-4598-9-200-S3.PDF]

‚Extracellular Region, Part'

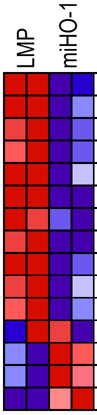

MMP2	matrix metallopeptidase 2 (gelatinase A, 72kDa gelatinase, 72kDa type IV collagenase)
TGFB1	transforming growth factor, beta 1 (Camurati-Engelmann disease)
ECM1	extracellular matrix protein 1
LGALS3BP	lectin, galactoside-binding, soluble, 3 binding protein
QSOX1	
SGCE	sarcoglycan, epsilon
SGCB	sarcoglycan, beta (43kDa dystrophin-associated glycoprotein)
SFN	stratifin
GPC1	glypican 1
COL9A3	collagen, type IX, alpha 3
FBLN1	fibulin 1
CFP	complement factor properdin
FKTN	
ERBB2IP	erbb2 interacting protein
LOXL2	lysyl oxidase-like 2

‚Extracellular Space'

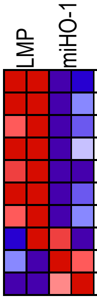

MMP2	matrix metallopeptidase 2 (gelatinase A, 72kDa gelatinase, 72kDa type IV collagenase)
TGFB1	transforming growth factor, beta 1 (Camurati-Engelmann disease)
LGALS3BP	lectin, galactoside-binding, soluble, 3 binding protein
QSOX1	
SFN	stratifin
GPC1	glypican 1
FBLN1	fibulin 1
CFP	complement factor properdin
FKTN	
LOXL2	lysyl oxidase-like 2

‚Receptor Binding'

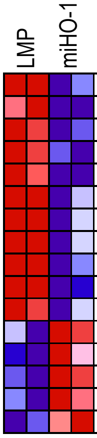

TGFB1	transforming growth factor, beta 1 (Camurati-Engelmann disease)
CLEC11A	C-type lectin domain family 11, member A
IGF2	insulin-like growth factor 2 (somatomedin A)
FYB	FYN binding protein (FYB-120/130)
TACC2	transforming, acidic coiled-coil containing protein 2
GDF15	growth differentiation factor 15
GRN	granulin
TRIP6	thyroid hormone receptor interactor 6
GAS6	growth arrest-specific 6
WIPI1	WD repeat domain, phosphoinositide interacting 1
PGF	placental growth factor, vascular endothelial growth factor-related protein
SRI	sorcin
NCK1	NCK adaptor protein 1
MED17	
ERBB2IP	erbb2 interacting protein
EFNB2	ephrin-B2

‚Cation Binding'

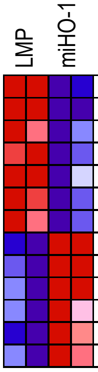

MMP2	matrix metallopeptidase 2 (gelatinase A, 72kDa gelatinase, 72kDa type IV collagenase)
CRIP2	cysteine-rich protein 2
ZNF185	zinc finger protein 185 (LIM domain)
GSN	gelsolin (amyloidosis, Finnish type)
CRIP1	cysteine-rich protein 1 (intestinal)
ATP7B	ATPase, Cu++ transporting, beta polypeptide
PRNP	prion protein (p27-30) (Creutzfeldt-Jakob disease, Gerstmann-Strausler-Scheinker syndrome)
GPD2	glycerol-3-phosphate dehydrogenase 2 (mitochondrial)
TIMM9	translocase of inner mitochondrial membrane 9 homolog (yeast)
RLF	rearranged L-myc fusion
EEA1	early endosome antigen 1, 162kD
ZNF146	zinc finger protein 146
MDM4	Mdm4, transformed 3T3 cell double minute 4, p53 binding protein (mouse)

‚Integral To Plasma Membrane‘

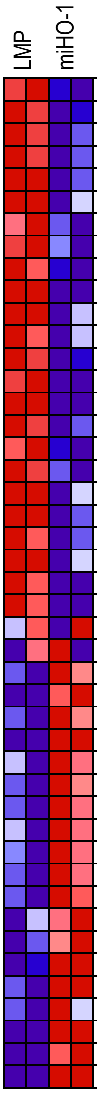

SLC7A1	solute carrier family 7 (cationic amino acid transporter, y+ system), member 1
TRPV2	transient receptor potential cation channel, subfamily V, member 2
ADAM8	ADAM metallopeptidase domain 8
ACCN2	amiloride-sensitive cation channel 2, neuronal
SLC1A4	solute carrier family 1 (glutamate/neutral amino acid transporter), member 4
SLC16A3	solute carrier family 16, member 3 (monocarboxylic acid transporter 4)
ACVR1B	activin A receptor, type IB
ADCY3	adenylate cyclase 3
SLC23A2	solute carrier family 23 (nucleobase transporters), member 2
FGFR3	fibroblast growth factor receptor 3 (achondroplasia, thanatophoric dwarfism)
FGFR4	fibroblast growth factor receptor 4
CAV2	caveolin 2
TSPAN15	tetraspanin 15
GRM4	glutamate receptor, metabotropic 4
SGCE	sarcoglycan, epsilon
ATP7B	ATPase, Cu++ transporting, beta polypeptide
NOTCH3	Notch homolog 3 (Drosophila)
SGCB	sarcoglycan, beta (43kDa dystrophin-associated glycoprotein)
TRIP6	thyroid hormone receptor interactor 6
GPC1	glypican 1
CSF3R	colony stimulating factor 3 receptor (granulocyte)
EBP	emopamil binding protein (sterol isomerase)
ITGB4	integrin, beta 4
PGRMC1	progesterone receptor membrane component 1
KCNQ2	potassium voltage-gated channel, KQT-like subfamily, member 2
GP9	glycoprotein IX (platelet)
ATP2B4	ATPase, Ca++ transporting, plasma membrane 4
SLC40A1	solute carrier family 40 (iron-regulated transporter), member 1
FRS2	fibroblast growth factor receptor substrate 2
HS3ST3B1	heparan sulfate (glucosamine) 3-O-sulfotransferase 3B1
CD47	CD47 molecule
MERTK	c-mer proto-oncogene tyrosine kinase
PTPRM	protein tyrosine phosphatase, receptor type, M
SLMAP	sarcolemma associated protein
KTN1	kinectin 1 (kinesin receptor)
IL1RAP	interleukin 1 receptor accessory protein
ST3GAL5	ST3 beta-galactoside alpha-2,3-sialyltransferase 5
ROR1	receptor tyrosine kinase-like orphan receptor 1
EFNB2	ephrin-B2
CLCNKA	chloride channel Ka
FZD3	frizzled homolog 3 (Drosophila)
MME	membrane metallo-endopeptidase (neutral endopeptidase, enkephalinase)
GOLM1	
SLC34A2	solute carrier family 34 (sodium phosphate), member 2
TNFSF10	tumor necrosis factor (ligand) superfamily, member 10

‚Transition Metal Ion Binding‘

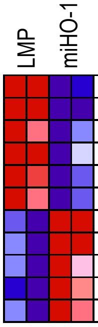

MMP2	matrix metallopeptidase 2 (gelatinase A, 72kDa gelatinase, 72kDa type IV collagenase)
CRIP2	cysteine-rich protein 2
ZNF185	zinc finger protein 185 (LIM domain)
CRIP1	cysteine-rich protein 1 (intestinal)
ATP7B	ATPase, Cu++ transporting, beta polypeptide
PRNP	prion protein (p27-30) (Creutzfeldt-Jakob disease, Gerstmann-Strausler-Scheinker syndrome)
TIMM9	translocase of inner mitochondrial membrane 9 homolog (yeast)
RLF	rearranged L-myc fusion
EEA1	early endosome antigen 1, 162kD
ZNF146	zinc finger protein 146
MDM4	Mdm4, transformed 3T3 cell double minute 4, p53 binding protein (mouse)

‚Ion Binding‘

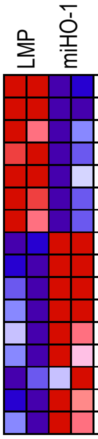

MMP2	matrix metallopeptidase 2 (gelatinase A, 72kDa gelatinase, 72kDa type IV collagenase)
CRIP2	cysteine-rich protein 2
ZNF185	zinc finger protein 185 (LIM domain)
GSN	gelsolin (amyloidosis, Finnish type)
CRIP1	cysteine-rich protein 1 (intestinal)
ATP7B	ATPase, Cu++ transporting, beta polypeptide
PRNP	prion protein (p27-30) (Creutzfeldt-Jakob disease, Gerstmann-Strausler-Scheinker syndrome)
STK38L	serine/threonine kinase 38 like
GPD2	glycerol-3-phosphate dehydrogenase 2 (mitochondrial)
TIMM9	translocase of inner mitochondrial membrane 9 homolog (yeast)
RLF	rearranged L-myc fusion
STK3	serine/threonine kinase 3 (STE20 homolog, yeast)
EEA1	early endosome antigen 1, 162kD
MT1X	metallothionein 1X
ZNF146	zinc finger protein 146
MDM4	Mdm4, transformed 3T3 cell double minute 4, p53 binding protein (mouse)

‚System Development‘

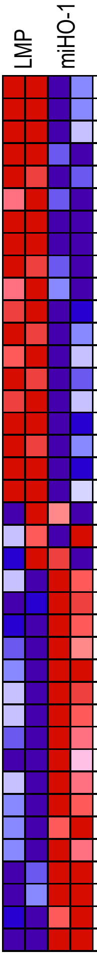

TGFB1	transforming growth factor, beta 1 (Camurati-Engelmann disease)
TPP1	tripeptidyl peptidase I
DPYSL5	dihydropyrimidinase-like 5
APOE	apolipoprotein E
IGF2	insulin-like growth factor 2 (somatomedin A)
ACVR1B	activin A receptor, type IB
FGFR3	fibroblast growth factor receptor 3 (achondroplasia, thanatophoric dwarfism)
SGCE	sarcoglycan, epsilon
SGCB	sarcoglycan, beta (43kDa dystrophin-associated glycoprotein)
NRTN	neurturin
NRGN	neurogranin (protein kinase C substrate, RC3)
SOX15	SRY (sex determining region Y)-box 15
DHCR24	24-dehydrocholesterol reductase
PML	promyelocytic leukemia
DVL2	dishevelled, dsh homolog 2 (Drosophila)
COL1A1	collagen, type I, alpha 1
HDAC5	histone deacetylase 5
TAZ	tafazzin (cardiomyopathy, dilated 3A (X-linked); endocardial fibroelastosis 2; Barth syndrome)
EBP	emopamil binding protein (sterol isomerase)
GHRHR	growth hormone releasing hormone receptor
KCNQ2	potassium voltage-gated channel, KQT-like subfamily, member 2
POU6F1	POU domain, class 6, transcription factor 1
NRD1	nardilysin (N-arginine dibasic convertase)
EXT1	exostoses (multiple) 1
NCOA4	nuclear receptor coactivator 4
NCKAP1	NCK-associated protein 1
COL12A1	collagen, type XII, alpha 1
SRI	sorcin
UBE3A	ubiquitin protein ligase E3A (human papilloma virus E6-associated protein, Angelman syndrome)
RTN4	reticulon 4
IQCB1	IQ motif containing B1
DSP	desmoplakin
FKTN	
ENC1	ectodermal-neural cortex (with BTB-like domain)
UTRN	utrophin (homologous to dystrophin)
CASP14	caspase 14, apoptosis-related cysteine peptidase
CACNB2	calcium channel, voltage-dependent, beta 2 subunit
HEY1	hairy/enhancer-of-split related with YRPW motif 1
ALX1	

‚Membrane Fraction‘

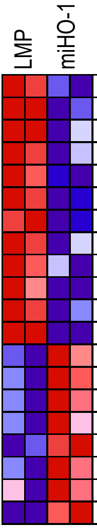

HMOX1	heme oxygenase (decycling) 1
SLC1A4	solute carrier family 1 (glutamate/neutral amino acid transporter), member 4
SLC16A3	solute carrier family 16, member 3 (monocarboxylic acid transporter 4)
A4GALT	alpha 1,4-galactosyltransferase (globotriaosylceramide synthase)
SLC23A2	solute carrier family 23 (nucleobase transporters), member 2
TSPAN15	tetraspanin 15
PDE4A	phosphodiesterase 4A, cAMP-specific (phosphodiesterase E2 dunce homolog, Drosophila)
SLC4A2	solute carrier family 4, anion exchanger, member 2 (erythrocyte membrane protein band 3-like 1
CYP19A1	cytochrome P450, family 19, subfamily A, polypeptide 1
CDH5	cadherin 5, type 2, VE-cadherin (vascular epithelium)
LASS5	LAG1 homolog, ceramide synthase 5 (S. cerevisiae)
ATP6V1F	ATPase, H+ transporting, lysosomal 14kDa, V1 subunit F
FRS2	fibroblast growth factor receptor substrate 2
ERO1L	ERO1-like (S. cerevisiae)
KTN1	kinectin 1 (kinesin receptor)
EEA1	early endosome antigen 1, 162kD
DSC3	desmocollin 3
UTRN	utrophin (homologous to dystrophin)
RPL7A	ribosomal protein L7a
SLC34A2	solute carrier family 34 (sodium phosphate), member 2
